# Measuring the Non-Linear Relationship between Three-Dimensional Built Environment and Urban Vitality Based on a Random Forest Model

**DOI:** 10.3390/ijerph20010734

**Published:** 2022-12-30

**Authors:** Jinyao Lin, Yaye Zhuang, Yang Zhao, Hua Li, Xiaoyu He, Siyan Lu

**Affiliations:** School of Geography and Remote Sensing, Guangzhou University, Guangzhou 510006, China

**Keywords:** three-dimensional built environment, urban vitality, vertical urban planning, machine learning

## Abstract

Urban vitality is a major indicator used for evaluating the sustainability and attractiveness of an urban environment. Global experience indicates that urban vitality can be stimulated through a reasonable urban design. However, it remains incompletely understood in the literature which building-related indicators can substantially affect urban vitality in Asian countries. To give an insight into this question, our study took a step forward by focusing specifically on the influence of the three-dimensional built environment on urban vitality, based on which decision makers could enhance urban vitality from the perspective of vertical building design. A machine-learning-based framework was developed in this study. First, we utilized several building-related indicators to thoroughly measure the spatial characteristics of buildings at the township level. Second, the relationship between a three-dimensional built environment and urban vitality was revealed based on a combined use of the correlation method, scatter charts, and a random forest. In the random forest, both a benchmark and a new model were constructed to evaluate the importance of those building-related indicators. The results suggested that urban vitality was closely related to the three-dimensional built environment, which played an even more important role than common benchmark factors in stimulating urban vitality. The building coverage ratio, density of tall buildings, and floor area ratio were essential spatial drivers behind urban vitality. Therefore, urban designers and decision makers should not only take traditional factors into account but also carefully consider the potential influence of high-rise buildings and the outdoor thermal environment so that urban vitality can be enhanced. Our study’s results can offer practical recommendations for improving urban vitality from the perspective of vertical building design. The proposed framework can also be used for measuring the potential influence of the three-dimensional built environment in other areas.

## 1. Introduction

While urban expansion is beneficial for socio-economic development, it can also cause various negative effects [1,2]. For example, an unreasonable urban development strategy and low-quality urban design will lead to the decline of urban vitality, both in old urbanized areas and newly developed areas [3,4,5]. In general, urban vitality refers to the spatial distribution, intensity, and frequency of people’s socio-economic activities [6,7,8]. In most cases, an improvement in urban vitality can enhance the sustainability and quality of people’s daily lives, such as public safety, social cohesion, and mental health [9,10,11]. To this end, an increasing number of regions have called for the design of vibrant and lively cities during the past decades. Numerous studies showed that urban vitality can be stimulated through reasonable urban planning and design [12,13,14].

In fact, the development of a vibrant urban environment greatly hinges on a thorough understanding of the key drivers behind urban vitality [15,16,17,18,19,20]. Although previous studies revealed the important relationship between the built environment (e.g., the size of built-up areas, urban form, urban growth pattern) and urban vitality, the conclusions were mainly drawn from a two-dimensional perspective. However, the built environment is not only reflected in the horizontal dimension but also the vertical dimension [21,22,23]. Specifically, the built environment relates to the artificial facilities and structures in which people live, work, and play, including streets, open spaces, and buildings [24,25,26,27,28].

Particularly in fast-growing regions, such as southeastern China, very limited land resources are still available for horizontal urban development after several decades of rapid expansion. Urban growth boundaries have also been established to prevent excessive encroachment on natural and agricultural lands. Therefore, the urban development strategy in highly-urbanized regions has been shifting from two-dimensional sprawl to three-dimensional expansion. In this regard, it is increasingly important to investigate the association between the three-dimensional built environment and urban vitality. To this end, our study will fill this knowledge gap by answering the following question: What is the overall impact of the three-dimensional built environment on urban vitality? The rest of this manuscript is organized as follows. In Section 2, a thorough literature review is given to further explain the importance of the three-dimensional built environment in urban vitality research. The details of the data and methods are introduced in Section 3. In Section 4, we present the results and associated policy implications. Lastly, concluding remarks are given in Section 5.

## 2. Literature Review

### 2.1. Proxy of Urban Vitality

Since urban vitality refers to the spatial distribution, intensity, and frequency of people’s socio-economic activities, the presence of people was commonly deemed as the proxy of urban vitality [29,30,31,32]. At first, people counting was mainly conducted based on field investigations and on-site interviews. For example, Zumelzu and Barrientos-Trinanes [14] measured the vitality level of a neighborhood using two on-site observation methods (gate method and static snapshot method). Mouratidis and Poortinga [9] conducted a two-month field survey to discover the conditions of urban vitality and social cohesion in a metropolitan city. Li et al. [33] estimated the number of pedestrians on twelve streets using street-view images. Delclòs-Alió and Miralles-Guasch [34] developed an urban vitality index that considers six dimensions (aggregation, variety, accessibility, communication opportunity, building history, and border vacuums) based on a local investigation. However, these field survey methods may not be desirable for measuring urban vitality at larger scales because they are usually labor-intensive and time-consuming.

Interestingly, the emergence of location-based services makes it possible to measure urban vitality based on “big data”. In particular, social media GPS tracking, mobile phone signaling data, social networking check-in records, and nighttime light data have been increasingly adopted for the assessment of urban vitality during the past decade. For example, both Fu et al. [35] and Dong et al. [36] used Tencent Location Big Data as the proxy of urban vitality over large regions. In addition, microblog check-in records were employed by Lu et al. [37] and Wu et al. [38] to represent urban vitality. Although urban vitality can be characterized in a more efficient way based on various types of “big data”, some limitations remain. For example, microblogs and other social networking applications are more popular among the younger generation. In addition, it is very difficult and costly to obtain complete mobile phone signaling data covering all major mobile network operators. Furthermore, nighttime light data can only roughly estimate the spatial distribution of population at night.

By contrast, the vitality information revealed by instant messaging applications should be a more suitable alternative. Particularly in China, instant messaging services are undoubtedly dominated by Tencent, one of the biggest Internet-based companies in the world. Therefore, this study adopted the Tencent Location Big Data, which can record the real-time number of people at a half-hour interval to quantify urban vitality. These location-based service data (with a spatial resolution of ~1000 m) were generated based on the massive deduplicated active users of various Tencent applications, such as WeChat, Tencent QQ, and Tencent Map [35,39]. Compared with traditional people counting, this information can effectively characterize the spatio-temporal distribution, intensity, and frequency of people’s daily activities [35,36,39].

### 2.2. Spatial Drivers behind Urban Vitality

Previous studies showed that urban vitality can be affected by accessibility, transportation condition, amenities, and land-use diversity [40,41,42,43,44]. For example, Long and Huang [45] analyzed the influence of urban form (e.g., intersection density, mixed land use, distance to transportation) on urban vitality and found that small blocks and higher levels of mixed land use can stimulate urban vitality. Yue et al. [46] examined the influence of POI (point of interest) mixed land use on neighborhood vitality and found that an increase in POI richness can greatly enhance neighborhood vitality. Mouratidis and Poortinga [9] pointed out that urban vitality will increase with basic amenities, distance to the urban center, and surrounding density, while it will decrease with the density of green space. He et al. [47] explored the influence of three urban sprawl patterns (i.e., edge, outlying, infilling) on urban vitality based on association rule analysis. Wu et al. [38] found that the number of POIs, distance to urban centers, and road density can exert substantial influences on urban vitality.

Recent studies also showed that urban vitality will be greatly affected by the characteristics of neighboring buildings. For example, Ye et al. [21] analyzed the influence of urban morphology (floor area ratio, ground space index, building height) on urban vitality and found that building typology is a more important driver than other factors in stimulating urban vitality. Xia et al. [48] focused on the influence of land development intensity (e.g., building coverage ratio) on urban vitality. Li et al. [49] revealed the impact of urban form on urban vitality and noted that building coverage ratio and building height have positive influences on urban vitality. Lu et al. [37] pointed out that the urban vitality in two Chinese cities is significantly affected by the building density and floor area ratio at the township level. Fan et al. [50] focused on the influence of the built environment on urban vitality along urban waterfronts. Their results showed that building density has the highest positive relationship with urban vitality. Yang et al. [51] analyzed the non-linear relationship between urban vitality and the built environment and found that floor area metrics play leading roles in predicting urban vitality. Lv et al. [52] suggested that the floor area ratio should be restrained within a suitable range and building density should be compatible with local development strategies so that urban vitality can be enhanced.

Although some of the above indicators already consider what takes place in the built environment vertically, the influences of building height and building configuration on urban vitality remain incompletely understood. It is still insufficient if urban vitality is measured only from a two-dimensional perspective because the spatial distribution characteristics of buildings will seriously affect the wind and thermal environment [53,54]. For example, an unreasonable building configuration can cause severe air pollution and increase the land surface temperature, thereby affecting the comfort level of people’s daily activities [55,56]. Although previous research proposed a dozen vertical spatial indicators (such as sky view factor, spatial congestion degree, tallest building height) to thoroughly exhibit the spatial characteristics of buildings [57,58,59], the association between these informative indicators and urban vitality remains to be further investigated. In particular, it remains incompletely understood in the literature which building-related indicators will play more important roles in stimulating urban vitality in Asian countries. Therefore, the importance of the three-dimensional built environment in urban vitality research will be specifically highlighted in this study.

### 2.3. Techniques Used for Analyzing the Drivers behind Urban Vitality

Generally speaking, the correlation analysis and ordinary least squares (OLS) linear regression model are the most popular methods for analyzing the spatial drivers behind urban vitality. However, the potential spatial effect of urban vitality cannot be considered in these two methods. To overcome this issue, some other useful techniques, such as the spatial lag model, the spatial error model, and geographically weighted regression, were also adopted by different studies [37,60]. While the above commonly-used methods are all intrinsically linear, an increasing number of studies pointed out that non-linear models would be more suitable in urban research because cities are complex systems [51,61].

Interestingly, a random forest is one of the most commonly used methods for tackling various non-linear mathematical problems [62,63]. While correlation analysis and spatial regression models are mainly used for linear research, the non-linear importance of each driver to urban vitality can be effectively evaluated using the random forest algorithm. More importantly, a random forest is insensitive to the multicollinearity issue between the variables. Considering the advantages and disadvantages of these different techniques, a combined use of correlation analysis, spatial regression, and a random forest may offer more rigorous outcomes than when each is employed separately.

## 3. Materials and Methods

**Figure 1:**
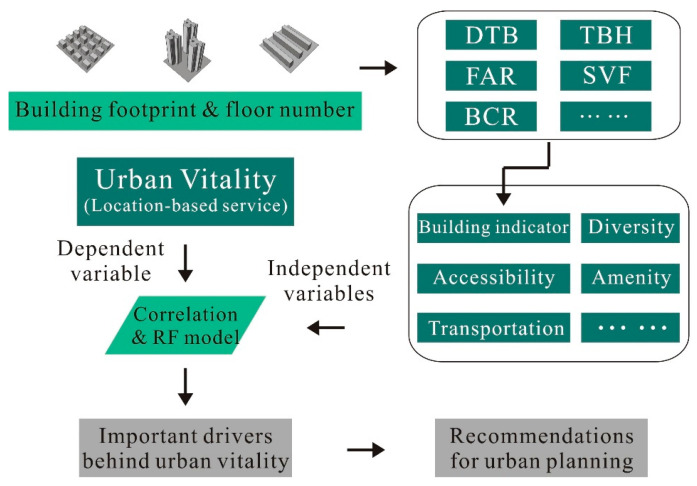
Methodological framework of this study.

This study aimed to investigate the integrated influences of the three-dimensional built environment and common benchmark factors on urban vitality, based on which decision makers could enhance urban vitality from the perspective of vertical building design. As illustrated in Figure 1, we first evaluated the spatial configuration of buildings with the support of various three-dimensional indicators. Second, urban vitality was quantified according to location-based big data. Third, both the linear correlation method and spatial regression models were used to measure the relationship between urban vitality and its potential drivers. Lastly, two experiments were conducted to further reveal the non-linear importance of every driver based on a random forest (RF) regression. The first one only took account of traditional drivers, whereas our proposed method also paid attention to three-dimensional indicators. The detailed methodology is presented in this section.

### 3.1. Data Collection and Processing

**Figure 2:**
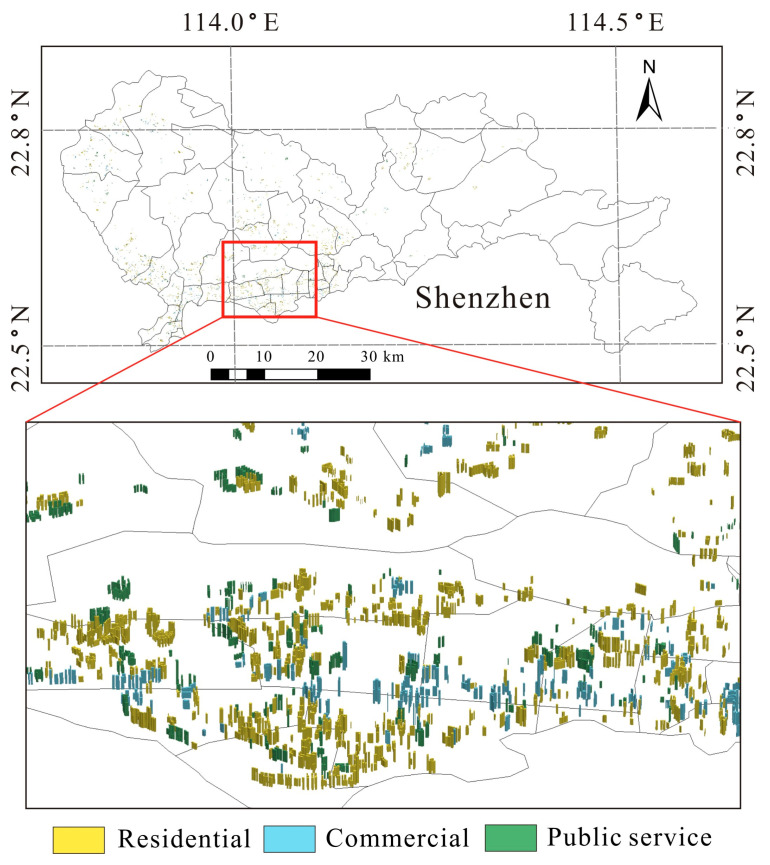
Location of Shenzhen and distribution of tall buildings.

A fast-growing high-density megacity (Shenzhen, China) was selected as our case study (Figure 2). Shenzhen, which has more than 140 high-rise buildings over 200 m, is the city with the highest number of skyscrapers in China. Although Shenzhen is one of the four first-tier cities in this country, attention should still be paid to the improvement of urban sustainability and quality of life. Thus, it is essential to identify the spatial drivers behind urban vitality so that urban vitality can be enhanced based on reasonable urban planning. The township (subdistrict) was adopted as the basic unit for investigation because it is also the basic unit for urban design and land-use planning. Planning recommendations cannot be put into practice easily if finer units (e.g., lattice and block level) are selected. Shenzhen includes 55 townships with different levels of social and economic development.

Based on the extensive literature review results given in Section 2, we used the Tencent Location Big Data, which can record the real-time number of people at a half-hour interval to quantify the urban vitality in Shenzhen. These location-based service data (with a spatial resolution of ~1000 m) were generated based on the massive deduplicated active users of various Tencent applications, such as WeChat, Tencent QQ, and Tencent Map. The data we collected included detailed population information from two consecutive weeks (6 July 2019–19 July 2019). In this study, the daily average accumulated population density at the township level was considered the dependent variable (i.e., urban vitality). In addition, point of interest (POI) data and building information in 2019 were obtained from Baidu Maps, which is one of the best companies offering navigation, mapping, and location-based services in China. POI data contain the category labels (e.g., residence, recreation), names, and coordinates of various geographical entities, while the building information includes the spatial footprint and floor number of every building. All these data were carefully corrected by removing the records that were duplicated or incomplete.

Previous research proposed a dozen vertical spatial indicators to thoroughly exhibit the spatial attributes of buildings [53,64]. As shown in Table 1, those indicators that can potentially affect urban vitality were adopted for this investigation. We did not rely on linear correlation methods for variable selection because a non-linear relationship most probably existed.

**Figure 3:**
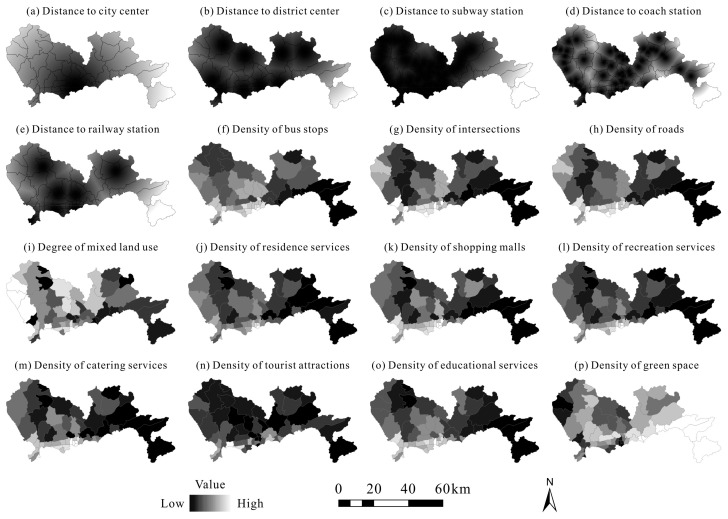
Benchmark spatial drivers behind urban vitality.

According to the literature review, urban vitality can also be affected by accessibility, transportation condition, amenities, and land-use diversity [41,65,66,67,68]. Therefore, several relevant indicators were considered as the benchmark for comparison (Table 2 and Figure 3). We first calculated the minimum distance to each accessibility driver at the city level using the “Euclidean Distance” tool in ArcGIS, and then the average distance score at the township level was regarded as an accessibility indicator in this study. In addition, land-use diversity can be quantified based on the degree of mixed land use (MIXED) [30,37,69]:

(1) $$\mathrm{MIXED}=-\sum_{i=1}^{n}\left(p_{i}\times \ln p_{i}\right)$$

where *p_i_* denotes the percentage of the *i*th POI category and *n* denotes the number of POI categories. In addition, the density of different POI categories (POID*_i_*) can be measured using.

(2) $${\mathrm{POID}}_{i}={\mathrm{POIN}}_{i}/A$$

where POIN*_i_* is the POI number of the *i*th category and *A* is the area at the township level.

### 3.2. Correlation Analysis

A correlation analysis can measure the linear association between two individual variables. A negative correlation coefficient suggests that one variable will increase as the other decreases and vice versa. The absolute value of coefficients can also reveal the strength of the linear relationship. In addition, scatter charts were adopted to further reveal the association between each building-related indicator and urban vitality.

### 3.3. Spatial Regression Models

Two widely-accepted spatial regression models (i.e., spatial lag model and spatial error model) were adopted in this study. First, multicollinearity was detected and corrected based on the variance inflation factor. Second, ordinary least squares regression was conducted in the GeoDa program to run the Lagrange multiplier test, which reveals whether the spatial dependence of urban vitality is significant [70]. The results obtained from ordinary least squares regression should be reserved for further analysis if neither the Lagrange multiplier lag nor the Lagrange multiplier error is statistically significant. Otherwise, robust Lagrange multiplier diagnostics were further employed to determine which spatial regression model was more suitable in this case. However, non-linear relationships most probably existed between different variables. Therefore, a more advanced method was also adopted to address the above disadvantages.

### 3.4. Random Forest (RF)

A random forest is a simple but effective ensemble-based machine learning algorithm that can address both regression and classification tasks [71]. The “forest” suggests that multiple decision trees are simultaneously constructed, while the “random” implies that both the samples and features for training each tree are randomly selected, which can prevent overfitting issues. A random forest uses the bagging technique, which is an ensemble method that trains each classifier based on different sub-datasets through replacement sampling on the original dataset. All the outputs from each classifier (i.e., decision tree) are combined by majority voting or averaging.

Numerous studies demonstrated that the combined model can generate a desirable result with higher accuracy and generalization ability. In addition, a random forest is insensitive to multicollinearity issues between the variables. More importantly, the permutation importance of each independent variable can be detected during the training process. Specifically speaking, the values for each independent variable are randomly shuffled and the associated difference in the model’s performance is measured. More important variables are generally more sensitive to such a permutation procedure. Therefore, the independent variables that substantially affect the performance should be considered more important to the dependent variable. The random forest algorithm has become one of the most commonly used methods for tackling various mathematical problems [62,72]. Therefore, this model was adopted to reveal the relationship between the three-dimensional built environment and urban vitality. Random forest models can be built according to WEKA (Waikato Environment for Knowledge Analysis) platform [73]. WEKA is open-source and easy-to-use software that is equipped with numerous kinds of machine learning methods.

In this study, we used a ten-fold cross-validation method to evaluate the performance of random forest models. In ten-fold cross-validation, all the samples (i.e., data at the township level) will be randomly partitioned into ten subsets with almost equal sizes. One of these ten subsets was considered the validation set at each time, whereas all the rest were used as the training set. This procedure was repeated ten times, with each subset used exactly once as the validation set [74]. Lastly, we assessed the urban vitality values obtained via cross-validation according to actual data. We adopted three indicators (i.e., root relative square error, relative absolute error, and coefficient of determination) to evaluate the validation accuracies of the models.

## 4. Implementation and Results

### 4.1. Association between Each Independent Variable and Urban Vitality

**Figure 4:**
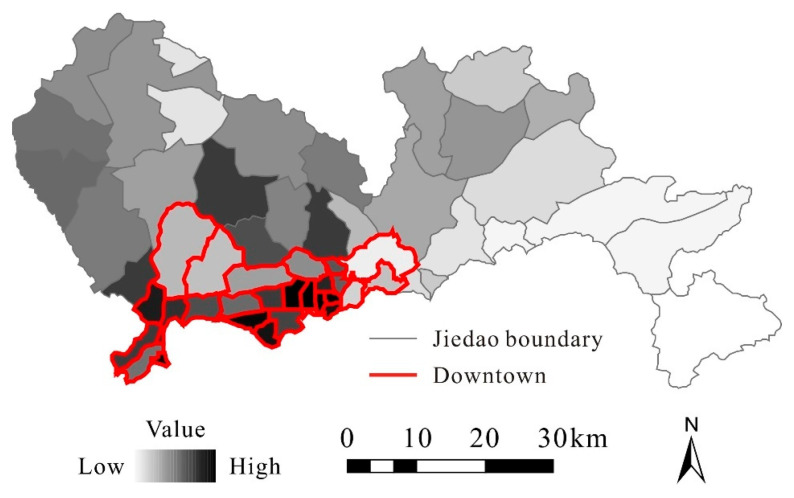
Daily average accumulated population density at the township level.

Figure 4 presents the daily average accumulated population density (urban vitality) at the township level. We found that the population density was generally higher in the downtown regions (i.e., Futian, Luohu, and Nanshan Districts) than in the outlying regions. This phenomenon is consistent with the social and economic conditions of this city, which suggests that the Tencent Location Big Data could effectively characterize the degree of urban vitality. We also found that the urban vitality still varied substantially between old urbanized areas and newly developed areas. In addition, there also existed some differences between the above three major districts. Thus, it was essential to figure out the spatial drivers behind urban vitality.

To this end, we first measured the linear correlation between each independent variable and dependent variable. In this study, the independent variables included 17 benchmark indicators and 15 building-related indicators, while the dependent variable was the daily average accumulated population density at the township level. As presented in Table 3, the correlation result indicated that BCR, FAR, the density of educational services, catering services, and shopping malls were the five most important drivers behind urban vitality. Moreover, many other building-related indicators (e.g., SCD, DTB, SVF, ABN) also exerted a positive contribution to urban vitality (1% significance level), while a negative relationship was found between urban vitality and the density of green space and the distance to a coach station and the city center (1% significance level).

It should be noted that there was a slight mismatch between the census population density and the dependent variable. This phenomenon could be attributed to two main reasons. First, the census reflects the population information only relating to residence. Therefore, human activities relating to other aspects were overlooked. Second, while the floating population and tourists are very important to urban vitality, they were not counted in the census.

**Figure 5:**
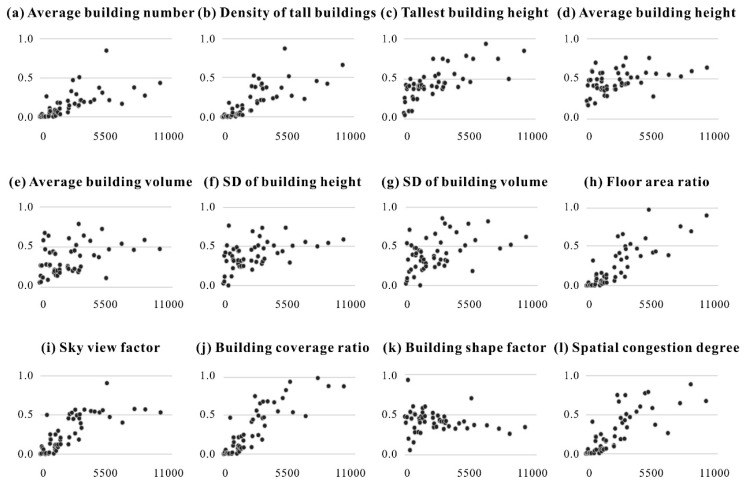
Scatter charts between urban vitality and each building-related indicator (the horizontal axis reflects the daily average accumulated population density at the township level, unit: people/km^2^; the vertical axis reflects the normalized score for each indicator).

Second, scatter charts were adopted to further reveal the complex association between urban vitality and various building-related indicators (Figure 5). The results suggested that the degree of urban vitality was enhanced as the BCR, FAR, SCD, DTB, and SVF increased. In addition, although urban vitality also generally increased with the TBH and ABH, some regions with higher TBH and ABH still exhibited a relatively lower vitality degree. These phenomena probably occurred because the decision makers blindly decided upon the height of tall buildings. Unfortunately, inappropriate planning of tall buildings will not necessarily promote competitiveness and attractiveness at the township level.

### 4.2. Association between All Independent Variables and Urban Vitality

The well-known GeoDa software program was used to examine whether the spatial dependence of urban vitality in Shenzhen is statistically significant. First, multicollinearity was detected and corrected based on the variance inflation factor using the SPSS package. Next, the remaining variables were input into the GeoDa, and the resultant Lagrange multiplier diagnostics indicated the absence of spatial dependence because neither the Lagrange multiplier lag (0.49705) nor the Lagrange multiplier error (0.32393) was statistically significant. Therefore, non-spatial regression methods were strong enough for our purpose.

Then, random forest regression was utilized to reveal the non-linear association between all the independent variables and urban vitality. Both a benchmark and a new model were constructed using WEKA to evaluate the importance of those building-related indicators. The latter used all the independent variables to explain urban vitality (Table 1 and Table 2), while the former excluded the building-related indicators. Note that the multicollinearity test was no longer necessary, and all variables were added to the final model in the random forest regression.

Table 4 presents the performance of the two random-forest-based models. We compared the difference between these two models in order to further highlight the necessity of considering these building-related indicators. We found that the benchmark model yielded a coefficient of determination (*r*^2^) of 0.7076, which suggested that 70.76% of the variation in urban vitality could be explained by the benchmark spatial factors. More importantly, 73.22% of the variation in urban vitality could be explained by the new model. Although there was no guarantee that the addition of new independent variables could enhance the explanation, these building-related variables in the second model still made a slight improvement. In addition, only 68.59% of the variation in urban vitality could be explained by the building-related model. These results suggested that although the three-dimensional built environment was not the dominant driver, the consideration of the three-dimensional built environment could still better characterize the degree of urban vitality. Therefore, the influence of the three-dimensional built environment should not be overlooked. Furthermore, the other two indicators (i.e., RAE and RRSE) indicated that the urban vitality scores obtained from the new model were closer to the ground truth data.

**Figure 6:**
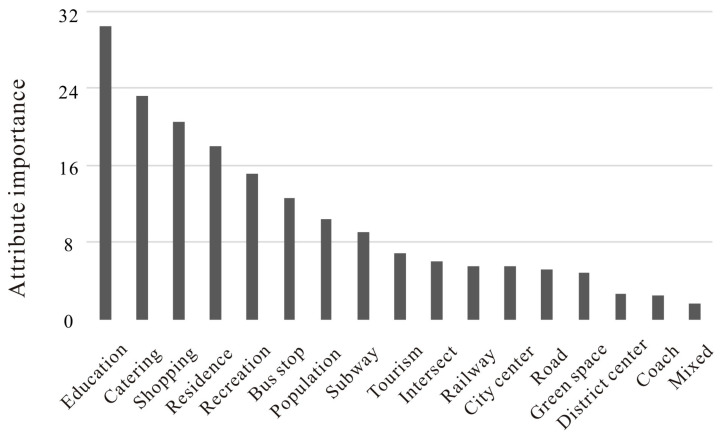
Permutation importance of every potential driver to urban vitality (benchmark model).

**Figure 7:**
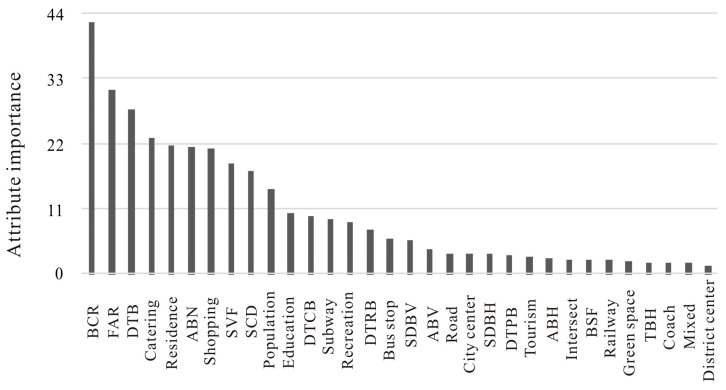
Permutation importance of every potential driver to urban vitality (new model).

Finally, the permutation importance of every potential driver to the degree of urban vitality is displayed in Figure 6 and Figure 7. A comparison of importance values better highlighted the huge influence of the three-dimensional built environment, which played an even more important role than common benchmark factors in stimulating urban vitality. Specifically, we found that the density of educational services, catering services, shopping malls, residence services, and recreation services were the variables that exerted the highest influence on urban vitality in the benchmark model. However, more importantly, the degree of urban vitality was more sensitive to the building coverage ratio, the density of tall buildings, and the floor area ratio in the new model. In addition, the average number of buildings, sky view factor, and spatial congestion degree could also make an important contribution to urban vitality. In summary, our experiments indicated that the three-dimensional built environment had a huge influence on urban vitality.

## 5. Discussion and Implications

While previous research pointed out the dramatic influence of the built environment on urban vitality, our study took a step forward by focusing specifically on building height and building configuration. In particular, it remains incompletely understood in the literature which building-related indicators will play more important roles in stimulating urban vitality. This study gave insight into answering this question. Specifically speaking, we investigated the integrated influence of various spatial factors through advanced machine learning methods. Compared with the mere consideration of benchmark indicators, the inclusion of the three-dimensional built environment could better explain the variation in urban vitality.

First, several conventional spatial factors, such as the density of residence services, catering services, shopping malls, and the distance to a subway station, could have profound impacts on the degree of urban vitality. These phenomena were consistent with the conclusions drawn from previous related research [13,30,38]. However, our results further suggested that urban designers and decision makers should not merely take into account these common factors. For example, the differences in amenities and transportation conditions among different regions tend to be relatively small ever since the nationwide promotion of “15-min life circle” programs in China [75]. Therefore, citizens and tourists may not necessarily be attracted if only the amenities and transportation conditions are improved.

Second, the density of tall buildings will exert a major influence on urban vitality. In fact, the quantity of high-rise landmarks and the size of the tallest buildings have been considered essential “signals” for social and economic conditions in numerous rapidly-urbanizing regions [76,77]. In general, places that consist of multiple high-rise buildings are extensively urbanized and well-developed parts of a city. These regions will not only be appealing to plenty of both residents and tourists but also attract large amounts of business and commercial investment. As a consequence, more high-quality job opportunities would be available for employees.

Third, the floor area ratio and spatial congestion degree, which are important indicators of urban development intensity, also played fundamental roles in urban vitality. A higher value of these metrics is strongly associated with the concentration of socio-economic activities (e.g., job opportunities, public facilities), which could accommodate a large number of people [72,78]. In addition, an increase in the floor area ratio and spatial congestion degree may potentially result in a higher housing supply and lower house prices at the regional scale, and thus, more residents will choose to settle down. It should also be noted that the blind and singular pursuit of high-density development may lead to the phenomenon of a “ghost town”. Therefore, all the key building-related and benchmark factors should be simultaneously considered to prevent this phenomenon from happening.

Fourth, our results also indicated that the building coverage ratio and average building number were important to urban vitality. This was probably because the hustle and bustle of urban life are in the possession of buildings. An increase in building coverage ratio and average building number will potentially attract more people to stay around. In contrast, passengers may experience a sense of desolation and emptiness in urban areas with limited building coverage (excluding urban parks) [63].

Fifth, the sky view factor, which determines the net heat storage in an urban microclimate, also played a role in urban vitality. This was probably because a lower fraction of visible sky can alter the land surface temperature [79,80,81], and thus, the comfort level of people’s daily activities in summer may also be affected.

Sixth, the densities of both tall residential and commercial buildings were important to urban vitality. However, the density of tall buildings still played a more substantial role. This phenomenon suggests that an uneven distribution of residential and commercial buildings may not enhance urban vitality. In addition, the public service buildings include administrative, educational, medical, sports, and cultural usages. Although the density of tall public service buildings also exerted a positive contribution to urban vitality, its influence was less than that of the other building-related indicators. This is because these kinds of buildings are usually attractive to only some specific groups of people.

Lastly, our results indicated that the standard deviation for building height and building volume, along with average building height, did not seriously affect the degree of urban vitality, which suggests that the dispersion and variability of building height and volume did not determine the intensity and frequency of people’s socio-economic activities.

Our results were also compared with previous studies on urban vitality in Shenzhen. Tang et al. [13] explored the influence of urban form on urban vitality in Shenzhen according to geographically and temporally weighted regression. They found that the influences of commercial and leisure functions on urban vitality were positive in most areas, but the distances to the city center and airport showed a negative correlation with vitality. Chen et al. [19] examined the relationship between the built environment and urban vitality in Shenzhen based on ordinary least squares regression. They found that the population density, POI indicators, and subway stations were substantially associated with urban vitality in Shenzhen. Ye et al. [21] analyzed the influence of urban morphology on urban vitality in Shenzhen and pointed out that building typology (floor area ratio, building height) is a more important driver than other factors in stimulating urban vitality. Li et al. [49] revealed the impact of urban form on urban vitality in Shenzhen based on a geographically and temporally weighted regression model and geographical detector. They noted that building coverage ratio and building height have positive influences on urban vitality. Yang et al. [51] analyzed the non-linear relationship between urban vitality and the built environment in Shenzhen and found that floor area metrics play leading roles in predicting urban vitality. In summary, previous conclusions on benchmark factors (i.e., accessibility, amenity, transportation condition, and common building-related factors) are generally in accordance with our findings. Regarding the three-dimensional built environment, however, previous studies merely considered the building height, floor area ratio, building coverage ratio, and building density. Therefore, this study filled the knowledge gap by paying more attention to various new building-related indicators.

Based on the above results and discussion, decision makers should consider the internal logic to stimulate urban vitality. First, several high-rise buildings could be properly developed and promoted as regional “landmarks” to attract business and commercial investment. Second, a high-density building configuration should be designed to ensure social and economic prosperity at the regional scale. Third, an uneven distribution of medium-rise and low-rise buildings may prevent the feeling of “aesthetic fatigue” among residents and tourists. Fourth, our results could also draw decision makers’ attention so that the sky-view condition of buildings could be taken into account at an early stage of urban design. The above configuration could not only provide sufficient space for living, working, and playing, but also guarantee the comfort level of pedestrians. It should also be pointed out that consideration of the three-dimensional built environment alone is insufficient to stimulate urban vitality. Decision makers still need to pay enough attention to the other traditional drivers, including amenities and accessibility. These findings may give an insight into urban design in three-dimensional space. Our policy recommendations should be properly considered by urban designers and decision makers when making three-dimensional regional planning.

Even though the proposed quantitative framework was only tested in a city, it could also be utilized for measuring the relationship between the three-dimensional built environment and urban vitality in various other regions with limited land resources, where vertical urban growth plays a fundamental role. Nonetheless, our study still contained some limitations that should be considered in future studies. First, the data collected by our study only included population information for two consecutive weeks due to data availability. We will try to obtain more data so that the temporal influence of the three-dimensional built environment can be measured. We could also test whether the association between the three-dimensional built environment and urban vitality changes at different times of the day in future studies. Second, the age of buildings was not considered since this information was also difficult to obtain. Fortunately, the impact of building age may not be significant because our study area has not been substantially developed since the 1990s. Third, the results should be generalized cautiously for non-Chinese regions because of the differences in the social, political, and built environment. Fourth, the relationship between the built environment and urban vitality was measured only at the township level because the township is the basic unit for urban design and land-use planning. The scale effect of the three-dimensional built environment on urban vitality should be further examined. Fifth, we did not consider the potential synergy between the independent variables in this study, which should be done in the future. Lastly, the building–street relationship was not examined in this study area. This was because many of the low-rise buildings in Chinese cities were distributed around urban villages, where people may feel unsafe and fearful of crime. However, the connections between buildings and streets are still strong. These phenomena are quite different from the conditions in American and European cities.

## 6. Conclusions

An improvement in urban vitality can substantially enhance the sustainability and quality of people’s daily lives. It is fundamental to fully interpret the spatial drivers of urban vitality, based on which urban designers and decision makers could stimulate urban vitality through reasonable spatial planning. However, previous studies mainly put emphasis on the relationship between the built environment and urban vitality in two-dimensional space. To overcome the above limitation, our study used a machine-learning-based method to thoroughly investigate the spatial drivers behind urban vitality.

Results have suggested that urban vitality is closely related to the three-dimensional built environment, which plays an even more important role than common benchmark factors in stimulating urban vitality. The comparison indicated that the building coverage ratio, the density of tall buildings, and floor area ratio, rather than the distance to a transportation network or urban center, were the leading generators of urban vitality in Shenzhen, followed by the density of residence services, catering services, and shopping malls.

In conclusion, urban designers and decision makers should not only take into account the floor area ratio, building coverage ratio, or some other traditional factors but also carefully consider the huge influence of high-rise buildings and the outdoor thermal environment so that urban vitality can be enhanced.

## Figures and Tables

**Table 1:** Information about three-dimensional spatial indicators.

Indicator	Calculation	Description
Average building number (ABN)	ABN=M/A	-
Density of tall buildings (DTB)	$DTB=M_{T}/A$	DTB is associated with the number of buildings with over 10 floors.
Density of tall residential buildings (DTRB)	-	-
Density of tall commercial buildings (DTCB)	-	-
Density of tall public service buildings (DTPB)	-	-
Tallest building height (TBH)	$TBH=\max\left(H_{i}\right)$	TBH is a dominance measure of building height.
Average building height (ABH)	$ABH=\sum_{i=1}^{M}H_{i}/M$	-
Average building volume (ABV)	$ABV=\sum_{i=1}^{M}V_{i}/M$	-
Standard deviation for building height (SDBH)	$SDBH=\sqrt{\frac{\sum_{i=1}^{M}{\left(H_{i}-ABH\right)}^{2}}{M}}$	SDBH indicates the dispersion and variability of building height relative to its mean.
Standard deviation for building volume (SDBV)	$SDBV=\sqrt{\frac{\sum_{i=1}^{M}{\left(V_{i}-ABV\right)}^{2}}{M}}$	SDBV indicates the dispersion and variability of building volume relative to its mean.
Floor area ratio (FAR)	$FAR=\frac{\sum_{i=1}^{M}\left(F_{i}*A_{i}\right)}{A}$	FAR indicates the relationship between gross floor area and the area of the lot on which the buildings sit.
Sky view factor (SVF)	$SVF=\int_{\theta =0}^{2\pi }\cos^{2}(\beta (R,\theta ))d\theta$	SVF quantifies the fraction of the surface radiation emitted by a planar surface to the radiation received by the whole sky hemisphere.
Building coverage ratio (BCR)	$BCR=\sum_{i=1}^{M}A_{i}/A$	BCR indicates the relationship between total footprint areas and the area of the lot on which the buildings sit.
Building shape factor (BSF)	$BSF=\frac{\sum_{i=1}^{M}\frac{P_{i}*H_{i}+A_{i}}{V_{i}}}{M}$	BSF indicates the relationship between the surface area of a building and its total volume.
Spatial congestion degree (SCD)	$SCD=\frac{\sum_{i=1}^{M}V_{i}}{\max\left(H_{i}\right)*A}$	SCD indicates the relationship between total building volumes and the maximum volume of the lot on which the buildings sit.

Note: *M* denotes the number of buildings, *M*_T_ denotes the number of tall buildings, *F_i_* denotes the floor number for the *i*th building, *H_i_* denotes the height for the *i*th building, *A* denotes the area at the township level, *A_i_* denotes the floor area for the *i*th building, *V_i_* denotes the volume for the *i*th building, *β* denotes the angle from the center to the tallest height at a maximum distance equal to the search radius (*R*), and *P_i_* denotes the perimeter for the *i*th building. All the indicators are quantified at the township level.

**Table 2:** Information on benchmark spatial drivers behind urban vitality.

Category	Indicator	Category	Indicator
Accessibility	Distance to city center	Diversity	Degree of mixed land use
			Population density
	Distance to district center	Amenity	Density of residence services
	Distance to subway station		Density of shopping malls
	Distance to coach station		Density of recreation services
	Distance to railway station		Density of catering services
Transportation condition	Density of bus stops		Density of tourist attractions
	Density of intersections		Density of educational services
	Density of roads		Density of green space

**Table 3:** Linear correlation between urban vitality and each independent variable.

Distance to City Center	Distance to District Center	Distance to Subway Station	Distance to Coach Station	Distance to Railway Station	Density of Bus Stops	Density of Intersections
−0.494 **	−0.377 *	−0.447 **	−0.526 **	−0.477 **	0.733 **	0.738 **
**Density of roads**	**Degree of mixed land use**	**Density of residence**	**Density of shopping**	**Density of recreation**	**Density of catering**	**Density of tourism**
0.748 **	0.369 *	0.671 **	0.820 **	0.699 **	0.823 **	0.119
**Density of education**	**Density of green space**	**Population density**	**ABN**	**DTB**	**TBH**	**SDBH**
0.832 **	−0.665 **	0.753 **	0.675 **	0.769 **	0.495 **	0.466 **
**SDBV**	**ABH**	**ABV**	**SVF**	**FAR**	**SCD**	**BSF**
0.450 **	0.435 **	0.389 **	0.750 **	0.835 **	0.773 **	−0.213
**BCR**	**DTRB**	**DTCB**	**DTPB**			
0.847 **	0.663 **	0.727 **	0.425 **			

Note: The two marks “*” and “**” suggest the 5% and 1% levels of significance, respectively.

**Table 4:** The performance of the benchmark and new models.

	Coefficient of Determination	RAE	RRSE
Benchmark	0.7076	16.38%	19.84%
New	0.7322	14.66%	18.02%
Building-related	0.6859	17.70%	21.06%

## Data Availability

Restrictions apply to the availability of these data. Data was obtained from Tencent and are available from the authors with the permission of Tencent.
